# Transitions in Depressive Symptom Profiles Among Adolescents with Persistent Bullying Victimization: A Latent Transition Analysis

**DOI:** 10.3390/bs16071072

**Published:** 2026-06-30

**Authors:** Jiannan Zheng, Qi Li, Ruifeng Liu

**Affiliations:** 1 School of Physical Education, Hubei University, Wuhan 430062, China; hubu2024112945@163.com; 2 School of Physical Education, Central China Normal University, Wuhan 430079, China

**Keywords:** bullying victimization, depressive symptoms, latent transition analysis

## Abstract

**Background:** Although depressive symptoms are common among adolescents who experience school bullying victimization, these symptoms show heterogeneity and seriously affect individuals’ physical and mental health. Our current understanding of the latent classes of depressive symptoms and their transition characteristics over time among adolescents who persistently experience school bullying victimization remains limited. Therefore, it is necessary to use latent transition analysis to identify different depressive symptom patterns and further examine the longitudinal stability and transition trajectories of these patterns. **Methods:** This retrospective study included 785 adolescents who reported experiencing school bullying victimization at both time points (T1 and T2). Among them, 60.9% were boys, with a mean age of 13.92 ± 1.69 years at T1 and an age range of 11 to 19 years. Participants completed a two-wave survey over a 12-month period. Latent transition analysis (LTA) was conducted to identify latent profiles of depressive symptoms and examine transitions between profiles over time. In addition, physical activity, sleep duration, and substance use were examined as predictors of transitions between depressive symptom profiles. **Results:** The depressive symptom profiles of adolescents who persistently experienced school bullying victimization were classified into the Low Symptom group, Moderate Symptom group, and High Symptom group. The proportions of these groups were 58.4%, 30.9%, and 10.7% at T1, and 32.1%, 45.3%, and 22.6% at T2, respectively. Overall, the profiles showed either class stability or transitions toward more severe symptom groups, with the High Symptom group showing a 75.8% probability of remaining in the same class at T2. Physical activity, sleep duration, and substance use were all important predictors of depressive symptom transitions among adolescents who persistently experienced bullying victimization. Specifically, physical activity and sleep duration were important protective factors (*p* < 0.001), whereas substance use was a negative influencing factor (*p* < 0.001). **Conclusions:** Depressive symptoms among adolescents who persistently experience bullying victimization show dynamic transition characteristics, and health behaviors significantly influence symptom evolution. These findings suggest that interventions should focus on improving health behaviors to alleviate or prevent the worsening of depressive symptoms.

## 1. Introduction

As a common stressor among children and adolescents, school bullying has been increasingly confirmed to exert adverse effects on health outcomes (Östberg et al., 2018; Källmén & Hallgren, 2021; Sigurdson et al., 2015; Y. Wang et al., 2025; Zhao et al., 2024) and has attracted widespread attention from scholars in the field of global public health. Multiple lines of evidence from meta-analyses have consistently demonstrated that school bullying victimization is prevalent among adolescents and is highly associated with psychological maladjustment in victims (Ariani et al., 2025; Ye et al., 2023). This intentional and repetitive harmful behavior perpetrated by an individual or group against those who are relatively vulnerable physically or psychologically (Fraga et al., 2022) may generate persistent negative stress experiences, thereby interfering with adolescents’ psychological adaptation and emotion regulation processes through affecting stress-regulation functions related to the hypothalamic–pituitary–adrenal (HPA) axis (Adams et al., 2021; Chen et al., 2018). From the perspective of developmental psychopathology, such manifestations characterized by psychological maladjustment, difficulties in emotion regulation, and impaired psychosocial functioning often present during development as internalizing problem phenotypes centered on depression and anxiety (Klein et al., 2022; Murray et al., 2025).

Existing cross-sectional studies have provided preliminary evidence for the association between school bullying victimization and depressive symptoms among adolescents. For example, a cross-sectional study conducted among Malaysian students found that adolescents who experienced school bullying were more likely to exhibit depressive symptoms (Abd Razak et al., 2019). In addition, a cross-sectional survey of public school students in China also indicated that school bullying victimization was significantly associated with increased levels of depressive symptoms (Y. Yang et al., 2025). Furthermore, longitudinal studies have confirmed the association between school bullying victimization and the subsequent development of depressive symptoms in adolescents from a developmental perspective. For instance, a cohort study conducted in Denmark demonstrated that individuals who experienced school bullying during adolescence had a higher risk of developing depressive symptoms in adulthood compared with those who had not been bullied (Winding et al., 2020). Another cohort study based on the Fragile Families and Child Wellbeing Study (FFCWS) database further reported that exposure to school bullying was associated with higher depressive symptom scores (Ajibewa et al., 2025). Although existing studies have relatively fully revealed the relationship between school bullying victimization and depressive symptoms, our understanding of the internal heterogeneity of depressive manifestations among bullied adolescents remains limited. Existing studies commonly classify depression scale scores into categorical variables according to clinical cut-off values or directly use total scale scores to represent depression severity (Jiang et al., 2022; Muyiduli et al., 2024; Peng et al., 2025; Scaramutti et al., 2024; Y. Yang et al., 2024). Such approaches are useful for evaluating the overall level of depressive symptoms, but they make it difficult to reveal differences among adolescents in specific symptom combinations (Thapar et al., 2022).

Latent class analysis/latent profile analysis provides a useful approach for identifying the heterogeneity of depressive symptoms. LCA/LPA can classify a sample into latent classes with distinct symptom characteristics according to individuals’ response probabilities across multiple symptom items (Ling et al., 2021). Previous studies have identified multiple classes of depressive symptoms in different populations. For example, an LCA conducted in a sociological sample of the Chilean population classified depressive symptoms into three classes: minimal, somatic, and severe (Carvajal et al., 2024). Another LCA based on a large sample of the Chinese population classified depressive symptoms into the “lack of positive affect” class, “low depressive symptoms” class, “high negative symptoms” class, “high depressive symptoms” class, and “possible clinical depression” class (Pan et al., 2024). These findings suggest that depressive symptoms are not expressed merely as a single continuum of severity but may involve distinct latent symptom patterns.

However, depressive symptoms in adolescents are developmental and dynamic in nature (M. Li et al., 2025). Especially among high-risk adolescents who experience school bullying, bullying victimization is not always a transient or occasional event. Existing studies have shown that both bullying perpetration and victimization tend to show considerable persistence, with victimization being more persistent than perpetration (Sourander et al., 2000). Adolescents who experience chronic bullying are more likely to develop multiple mental health adjustment problems and adverse health outcomes in adulthood (Giuliani et al., 2025). Therefore, compared with general adolescents, the developmental trajectories of depressive symptoms among bullied adolescents may be more likely to present unfavorable patterns, such as persistently high levels, gradual deterioration, or repeated fluctuations. However, existing studies still lack a systematic investigation of the patterns of symptom change over time within this group.

Latent transition analysis (LTA), as a longitudinal extension of latent profile analysis, is based on the Markov assumption and can simultaneously characterize the types of latent profiles and their discrete transition processes. It estimates the probability that individuals transition from a latent status at one time point to a latent status at the next time point, thereby providing an effective framework for revealing the dynamic evolution of depressive symptom development (Nylund-Gibson et al., 2023). Existing longitudinal studies using LTA have confirmed that latent profiles of adolescent depressive symptoms show clear dynamic characteristics, with individuals potentially transitioning among different depressive states, such as low, moderate, and high states (Dai et al., 2025; G. Li et al., 2023; Liang et al., 2023). However, most existing LTA studies have focused on general adolescent populations, with insufficient attention to transition patterns of depressive symptoms among adolescents who persistently experience school bullying victimization. Therefore, it is necessary to further examine the latent states of depressive symptoms and their cross-time transition patterns among adolescents who experienced school bullying victimization at both T1 and T2.

In addition to the high-risk context of persistent bullying victimization, individual behavioral characteristics and adaptive resources may jointly influence changes in adolescents’ depressive symptom states (Rogosch & Cicchetti, 2002). For adolescents who persistently experience bullying victimization, T1 health behavior factors, such as physical activity, sleep duration, and substance use, may reflect their baseline behavioral risks or protective resources and further influence subsequent transitions in depressive symptom states. On the one hand, higher levels of physical activity and adequate sleep are generally regarded as protective factors that contribute to emotion regulation and psychological recovery (Levková et al., 2025; K. Wang & Qiu, 2025). On the other hand, substance use may reflect maladaptive coping strategies and is associated with increased depressive symptoms (Kelly et al., 2015). Therefore, these T1 health behavior factors may influence the probabilities that adolescents who persistently experience bullying victimization transition from a lower depressive state to a higher depressive state, or remit from a higher depressive state to a lower depressive state, during the T1–T2 period.

However, research that integrates persistent bullying victimization and health behaviors and systematically examines their effects on the dynamic evolution of depressive profiles within an LTA framework remains limited. Based on the above background, this study focuses on adolescents who experienced school bullying victimization at both T1 and T2 and uses latent transition analysis to examine the latent states of their depressive symptoms and cross-time transition patterns. Specifically, this study aims to first, identify the latent states of depressive symptoms among adolescents who persistently experienced bullying victimization at T1 and T2; second, estimate the transition probabilities among different depressive states during the T1–T2 period; and third, examine the effects of T1 physical activity, substance use, and sleep duration on transition probabilities among depressive states. Based on previous studies, this study expects that latent states of depressive symptoms at low, moderate, and high levels may exist among adolescents who persistently experience bullying victimization. Given the chronic stress characteristics of persistent bullying victimization, transitions in depressive states among these adolescents may show a tendency toward deterioration. Higher levels of physical activity and longer or adequate sleep duration may be associated with improvement in depressive states, whereas substance use may be associated with deterioration in depressive states.

## 2. Method

### 2.1. Participants

This study was a retrospective longitudinal study conducted from September to November 2023 (T1) and from September to November 2024 (T2). The study was based on 30 surveillance sites for common diseases among students established across 17 prefecture-level cities and prefectures in Hubei Province. The surveillance sites were determined by local authorities under the guidance of a unified surveillance protocol, with comprehensive consideration of factors such as geographic location, urban–rural distribution, population size, school type, previous surveillance foundation, and feasibility of field investigation. These sites were intended to cover student populations from different regions and school types and therefore had a certain degree of regional representativeness. At each surveillance site, sample schools and students within the jurisdiction were selected using a stratified cluster sampling strategy. In principle, two junior high schools and two senior high schools were selected from the urban area of each prefecture-level city, while two junior high schools and one senior high school were selected from each county-level area. Within each school, grade was used as the stratification variable and class as the sampling unit. Whole classes were randomly selected from each grade, with at least 240 students selected from each school. If the number of students in a school was insufficient, supplementary samples were obtained from schools in the same region and of the same type to ensure that the sample size at each stratum met the survey requirements. All participating students were assigned a 16-digit unique identification number registered in the education system, which was used only for data archiving and matching. A total of 12,692 participants who completed the survey at both T1 and T2 were matched using the unique identification number. Bullying victimization was assessed using the revised Olweus Bully/Victim Questionnaire (Olweus, 1996). Initially, 799 participants who reported experiencing different forms of bullying behavior within the past 30 days at both T1 and T2 were included. We excluded 13 cases with missing information on the CES-D scale, physical activity, sleep duration, or substance use. Finally, 785 participants were included in this analysis. For cases with a small number of missing items on the CES-D scale, multiple imputation was performed separately at T1 and T2 using a cross-sectional imputation approach to make full use of the available information. Participants with missing demographic variables in the T2 questionnaire were retained in the analysis, and their T1 data were used to contribute information, thereby reducing sample bias and information loss.

Inclusion criteria: 1. Students who participated in both waves (T1 and T2) of the surveillance project on common diseases and health-related influencing factors; 2. Students who completed the questionnaires required for this study and had complete information on the main study variables and covariates. Exclusion criteria: 1. Participants with missing or incomplete information on major study variables, including depressive symptoms, physical activity, sleep duration, and substance use; 2. Participants whose questionnaire responses showed obvious logical errors or insufficient overall completion.

### 2.2. Ethics and Procedures

The study protocol was approved by the Ethics Committee of the Hubei Provincial Center for Disease Control and Prevention (Hubei Academy of Preventive Medicine) (Approval No. 2024-016-01) and complied with the ethical principles of the Declaration of Helsinki. Given that all participants in this study were minors, the research team obtained informed consent from the students’ parents/legal guardians and assent from the students themselves before data collection. Before the survey began, homeroom teachers explained the study purpose, study procedures, voluntary nature of participation, confidentiality measures, potential risks and benefits, and the fact that refusal to participate or withdrawal from the study would not have any adverse impact on the students to the students’ parents/legal guardians through a combination of online and offline methods. Parents/legal guardians provided consent orally or electronically. Before the formal start of the test, trained staff read the informed assent information to the students in a manner appropriate to their level of understanding and informed them that participation was entirely voluntary and that they could refuse to participate or withdraw at any time. Students participated in the test only after they indicated their willingness to participate. Data collection was conducted in classroom settings and was jointly organized and mutually supervised by homeroom teachers and professionally trained staff. Students independently completed the paper-based questionnaires. The ethics committee waived the requirement for written signatures; however, the procedures for informed consent and student assent were fully implemented. To protect participants’ privacy, each student was assigned a unique identification number for individual tracking across time points. Before the research team obtained the data, all information that could directly identify individuals had been removed or replaced, and the data were de-identified. The research team only accessed variable information relevant to the study purposes, and the research data did not contain any information that could directly identify individuals.

### 2.3. Measurement

#### 2.3.1. School Bullying

Bullying victimization status was assessed using six items from the revised Olweus Bully/Victim Questionnaire (Olweus, 1996). The questionnaire covers three types of bullying behaviors that occurred on or around campus within the past 30 days, including verbal bullying, relational bullying, and physical bullying. Items were rated on a 3-point scale (0 = never, 1 = occasionally, 2 = frequently). Participants who selected “occasionally” or “frequently” for any item were defined as bullying victims (Guo et al., 2010; Y. Yang et al., 2021). This scale has been widely used in studies among Chinese adolescents and has demonstrated good reliability and validity (Gong et al., 2019; Yan et al., 2018). In the present study, the Cronbach’s α coefficient was 0.81.

#### 2.3.2. Depressive Symptoms

Depressive symptoms were assessed using the Center for Epidemiologic Studies Depression Scale (CES-D) (Radloff, 1977). The scale consists of 20 items covering four dimensions: depressed affect (items 3, 6, 9, 10, 14, 17, and 18), positive affect (items 4, 8, 12, and 16), somatic symptoms (items 1, 2, 5, 7, 11, 13, and 20), and interpersonal problems (items 15 and 19). All items were rated on a 4-point scale (0 = less than 1 day, 1 = 1–2 days, 2 = 3–4 days, and 3 = 5–7 days). Items 4, 8, 12, and 16 were positively worded and were reverse-coded. Higher scores indicated more severe depressive symptoms. The scale has demonstrated good measurement properties in Chinese adolescent samples (Dou et al., 2021; W. Yang et al., 2018) and also showed high internal consistency in the present study, with a Cronbach’s α of 0.87.

#### 2.3.3. Physical Activity

Adolescents’ moderate-to-vigorous physical activity (MVPA) level was assessed using a self-reported single item. An example item was “During the past 7 days, on how many days did you engage in at least 60 min of moderate-to-vigorous physical activity for at least 60 min per day, accumulated across the day?” According to the recommended standard in the World Health Organization (WHO) Global Guidelines on Physical Activity for Adolescents (World Health Organization, 2020), engaging in moderate-to-vigorous physical activity on fewer than 3 days per week was classified as insufficient physical activity (0 = insufficient physical activity, 1 = sufficient physical activity).

#### 2.3.4. Substance Use

Based on the framework proposed by Pang et al. (2014), the substance use questionnaire was contextually adapted for the Chinese setting to assess participants’ lifetime and past use of various prescription medications and illicit substances without medical authorization. The questionnaire consisted of one multiple-choice item. An example item was “So far, have you ever used any of the following substances without a doctor’s permission? (Multiple answers are allowed.) The response options included (1) inhalable solvents such as glue, gasoline, and nitrous oxide; (2) cough syrup; (3) sedative–hypnotic medications, such as diazepam and triazolam; (4) tramadol; (5) cocaine; (6) pethidine; (7) morphine; (8) ecstasy; (9) methamphetamine; (10) ketamine; (11) fentanyl; (12) cannabis; (13) heroin; (14) opium; and (15) other substances (please specify). Participants who reported using any of the above substances without a doctor’s permission were defined as having substance use behavior and were coded as 1, whereas those who did not report any substance use were coded as 0. This questionnaire was used for descriptive classification and did not involve scale reliability analysis.

#### 2.3.5. Sleep Duration

According to the recommendations of the Consensus Statement on the Recommended Amount of Sleep for Pediatric Populations issued by the American Academy of Sleep Medicine (AASM) in 2016 (Paruthi et al., 2016), adolescents aged 13–18 years should regularly sleep for 8–10 h per 24 h. Sleep duration was assessed using a self-reported questionnaire item: “How many hours do you sleep each day?” Based on the questionnaire responses, sleep duration was dichotomously coded: 8–10 h was classified as “adequate sleep” and coded as 1, whereas less than 8 h was classified as “inadequate sleep” and coded as 0.

#### 2.3.6. Demographic Variables

Participants’ demographic information was collected using a self-administered questionnaire, including sex and age. Sex was coded as a categorical variable (1 = male, 2 = female), and age was recorded as actual age in years. Demographic variables were used only to describe sample characteristics and basic distributions and were not included as covariates or independent variables in subsequent statistical analyses.

### 2.4. Statistical Analysis

This study used latent transition analysis (LTA) to examine longitudinal changes in depressive symptoms among adolescents who persistently experienced school bullying victimization. LTA can be regarded as an extension of cross-sectional latent class/latent profile models (LCA/LPA) to longitudinal settings (Ghosh, 2010). At each time point, LTA estimates the class measurement model, including item-response probabilities for categorical indicators or within-class parameters for continuous indicators, as well as class proportions. Based on these estimates, it further estimates transition probabilities across time points, thereby describing individuals’ longitudinal transitions between latent states (Lee et al., 2025). This approach is suitable for studying behavioral and symptom changes that show stage-like patterns. Therefore, in the present study, latent transition analysis was used to identify individuals’ membership in different latent profiles based on depressive symptom indicators measured at two time points among adolescents who persistently experienced school bullying victimization and to characterize their profile transition patterns between adjacent time points. In addition, following the approach of Dai et al. (2025), covariates were incorporated into the mixture modeling framework using multinomial logistic regression to characterize transition probabilities conditional on covariates, as specified in the following formula:

$$P(C_{t}\;=\;j/C_{t-1}\;=\;i,\;X)$$

Using “remaining in the original state” (i.e., the reference category K = i) as the reference, the transition probabilities for j ≠ K were parameterized as follows:

$$\log\left(\frac{P\left(C_{t}\;=\;j\;|C_{t-1}\;=\;i,\;X\right)}{P\left(C_{t}\;=\;K\;|C_{t-1}\;=\;i,\;X\right)}\right)=\;\alpha_{ij}\;+\;\beta_{j}^{⊤X}$$

Model construction was performed using Mplus 8.3 (Muthén & Muthén, 2017), with the detailed steps as follows:

#### 2.4.1. Longitudinal Measurement Invariance Testing

To ensure that the meanings of the latent profiles were comparable between T1 and T2, across-time measurement invariance testing was conducted for the four dimensions of depressive symptoms. The tests were performed sequentially for configural invariance, metric invariance, scalar invariance, and error variance invariance. Measurement invariance was considered supported if the change in the comparative fit index (CFI) did not exceed 0.01 (Cheung & Rensvold, 2002).

#### 2.4.2. Latent Profile Analysis (LPA) and Parameter Selection

Using the mean scores of the four dimensions of the depressive symptom scale at T1 and T2 as analytical indicators, latent profile analysis (LPA) models were constructed. The optimal classification model was determined by comprehensively evaluating the sample size of each profile, the theoretical interpretability of the profiles, model parsimony, and multiple model fit indices. The indices considered included AIC (Akaike, 1974), BIC (Schwarz, 1978), aBIC (Bozdogan, 1987), entropy, VLMR (Vuong, 1989), and BLRT (McLachlan et al., 2019). Smaller AIC, BIC, and aBIC values indicated a more reliable model fit, whereas entropy values closer to 1 indicated higher classification accuracy (Schepers et al., 2020). When the p values of the VLMR and BLRT were less than 0.05, the k-class model was considered to have a better fit than the *k* − 1-class model. After determining the optimal number of latent profiles, the profile characteristics were visualized based on the posterior-probability-weighted means of the four dimensions of depressive symptoms in each profile, and the profiles were named accordingly (Băjenaru et al., 2022).

#### 2.4.3. Latent Transition Analysis (LTA) and Inclusion of Covariates

Based on the latent profiles constructed at the two time points, a longitudinal extension was performed to build the LTA model and examine individuals’ transition patterns among different profiles. By estimating the profile transition probability matrix, we analyzed whether participants were more likely to maintain their original depressive symptom profile membership at adjacent time points or transition to other profile types and thereby evaluated the temporal stability of profile membership. Regarding the strategy for covariate inclusion, this study adopted a time-lagged model, in which risk factors measured at the previous time point were used to predict subsequent latent profile transitions. This strategy helps avoid mistakenly treating post-transition manifestations as the basis for distinguishing risk within the model, thereby improving the interpretive rationality of transition prediction for risk stratification and, to some extent, reducing the influence of reverse causality bias. Therefore, sleep duration, physical activity, and substance use were included as covariates in the analysis of transition probabilities of participants’ latent profiles between T1 and T2 using a multinomial logistic regression model.

## 3. Results

### 3.1. Sample Characteristics

Table 1 presents the descriptive statistical results for participants’ depressive symptom profile characteristics, demographic characteristics, and health behaviors. The depressive symptom-related indicators at T1 and T2 were all significantly positively correlated with each other. Substance use was significantly positively correlated with depressive symptom indicators at both T1 and T2 and was significantly negatively correlated with physical activity and sleep duration. Correspondingly, both physical activity and sleep duration were significantly negatively correlated with depressive symptom indicators at the two time points, and physical activity was significantly positively correlated with sleep duration.

### 3.2. Longitudinal Measurement Invariance Across Time

As shown in Table 2, metric invariance and scalar invariance of the depressive symptom scale were supported. Although error variance (strict) invariance also showed ΔCFI < 0.01, considering conventional evaluation criteria (Little, 2024), metric and scalar invariance were sufficient to meet the core requirements of the present study.

### 3.3. Construction of Latent Depressive Symptom Profiles at Two Time Points

Latent profile analysis (LPA) was conducted separately at T1 and T2 to identify depressive symptom profiles, and the model fit indices for each time point are presented in Table 3. The results showed that as the number of profiles increased, the Akaike information criterion (AIC), Bayesian information criterion (BIC), and adjusted Bayesian information criterion (aBIC) all showed a continuous downward trend. At T1, when the number of profiles exceeded four, the improvement in model fit weakened, and the VLMR test became non-significant (*p* > 0.05). A similar pattern was observed at T2 when the number of profiles exceeded three (*p* > 0.05), indicating that further increasing the number of profiles did not significantly improve model fit. The entropy values of all candidate models at both time points were greater than 0.80, indicating acceptable classification accuracy. Further considering model interpretability and class distribution, at T1, although the 2-, 3-, and 4-profile models all showed acceptable fit indices, the 2-profile model provided insufficient differentiation of individual heterogeneity, whereas in the 4-profile model, the smallest class accounted for only 3.9% of the total sample, suggesting a risk of overfitting. At T2, both the 2-profile and 3-profile models were considered candidate models, among which the 3-profile model performed better in terms of AIC, BIC, aBIC, and entropy. Therefore, based on both statistical indices and theoretical interpretability, the 3-profile model was ultimately identified as the optimal model at both T1 and T2.

**Figure 1:**
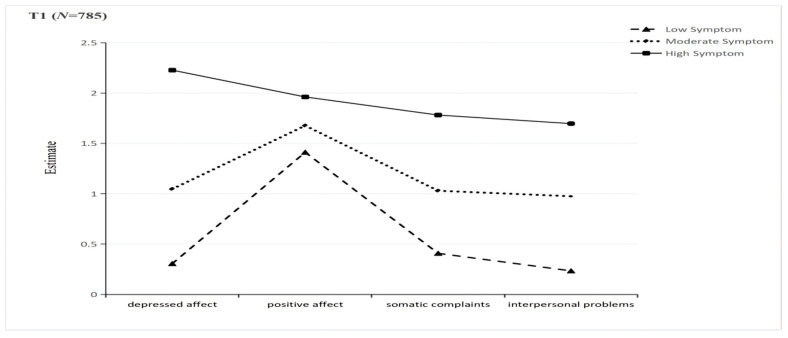
Potential profile of adolescents at time point T1.

**Figure 2:**
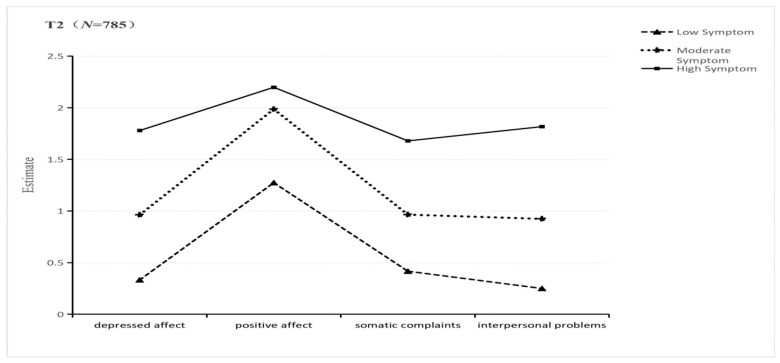
Potential profile of adolescents at time point T2.

Table 4 presents the estimated results for the latent depressive symptom profiles at the two time points. The results showed that the first class had the lowest mean scores on all four dimensions of depressive symptoms among the three classes, the second class had moderate mean scores on all dimensions, and the third class had the highest mean scores on all dimensions. The differences among the three classes on each dimension were all statistically significant. Based on these characteristics, the three profiles were named Low Symptom, Moderate Symptom, and High Symptom, respectively. The characteristic distributions of the latent profiles at the two time points are shown in Figure 1 and Figure 2.

### 3.4. Latent Status Changes Among Persistently Bullied Victims

Table 5 presents the modified unconditional latent class transition matrix for the T1–T2 interval. The initial unconditional transition matrix showed that only a very small number of cases transitioned from Class 1 at T1 to Class 3 at T2. Further examination of this parameter indicated that a transition probability of 0 between adjacent time points was theoretically reasonable; therefore, the parameter for this transition path was constrained to 0.

Specifically, the bold values on the diagonal of the transition matrix indicate the proportion of participants who remained in the same latent class across time points, whereas the off-diagonal values reflect transitions between latent classes; thus, the values in each row sum to 100%. The results showed that the High Symptom group had the strongest stability, with a retention rate of 75.8% during the T1–T2 interval. Only a small proportion of cases transitioned to the Moderate Symptom group and the Low Symptom group, with probabilities of 12.4% and 11.8%, respectively. In the Moderate Symptom group, 50.5% of cases remained in the same class at T2, 41.9% transitioned to the High Symptom group, and 7.6% transitioned to the Low Symptom group. In contrast, the Low Symptom group showed relatively weaker stability, with 51.2% of cases transitioning to the Moderate Symptom group at T2, while the remaining 48.8% stayed in the original class.

To further examine the effects of physical activity, substance use, and sleep duration on transitions over time among individuals belonging to different depressive symptom profiles, we used multinomial logistic regression analysis and incorporated covariates to characterize transition probabilities conditional on covariates. Detailed results are presented in Table 6. During the T1–T2 period, meeting the physical activity recommendation and meeting the sleep duration recommendation were both associated with more favorable latent class transition patterns. Using “remaining in the Low Symptom group” as the reference, individuals who met the physical activity recommendation and those who met the sleep duration recommendation had significantly lower likelihoods of transitioning to the Moderate Symptom group (OR = 0.166 and 0.131, respectively) and the High Symptom group (OR = 0.008 and 0.010, respectively) (all *p* < 0.001). Correspondingly, individuals with substance use behavior were more likely to transition from the Low Symptom group to the Moderate Symptom group or the High Symptom group (OR = 5.557 and 5.836, respectively; both *p* < 0.001).

Using “remaining in the Moderate Symptom group” as the reference, meeting the physical activity recommendation and meeting the sleep duration recommendation significantly reduced the likelihood of transitioning to the High Symptom group (OR = 0.048 and 0.077, respectively), and significantly increased the likelihood of transitioning to the Low Symptom group (OR = 6.026 and 7.652, respectively) (all *p* < 0.001). Meanwhile, individuals with substance use behavior had a significantly lower likelihood of transitioning from the Moderate Symptom group to the Low Symptom group (OR = 0.180, *p* < 0.001).

In addition, using “remaining in the High Symptom group” as the reference, meeting the physical activity recommendation and meeting the sleep duration recommendation both significantly increased the likelihood of transitioning from the High Symptom group to the Moderate Symptom group (OR = 21.031 and 12.988, respectively; both *p* < 0.001).

## 4. Discussion

Depressive symptoms are common mental health problems among children who experience bullying victimization, and victimization often shows persistence (Sourander et al., 2000). However, existing studies have paid relatively limited attention to the internal heterogeneity and dynamic developmental processes of depressive symptoms among children who persistently experience bullying victimization. Meanwhile, health behaviors such as physical activity, sleep duration, and substance use are considered to be closely associated with depressive symptoms in children and adolescents (Kelly et al., 2015; Levková et al., 2025; K. Wang & Qiu, 2025), but the roles of these factors in depressive symptom profile membership and its transition process remain insufficiently and systematically examined. Therefore, this study used latent transition analysis (LTA) to examine the latent profiles and transition patterns of depressive symptoms among children who persistently experienced bullying victimization, as well as the effects of physical activity, sleep duration, and substance use on symptom profile membership and transitions.

The main findings of this study were as follows: (1) depressive symptoms among children who persistently experienced bullying victimization showed significant heterogeneity, and latent profiles with different symptom levels could be identified; (2) the development of depressive symptoms showed overall strong class stability, while some children tended to transition toward more severe symptom profiles; and (3) meeting recommendations for physical activity and sleep duration may serve as protective factors in transitions of depressive symptom development among children who persistently experienced bullying victimization, whereas substance use may serve as a risk factor.

### 4.1. Profiles of Depressive Symptoms

This study identified three latent classes of depressive symptoms among children who persistently experienced bullying victimization. Specifically, the Low Symptom group, Moderate Symptom group, and High Symptom group were identified at both Time 1 (T1) and Time 2 (T2). This classification further verifies the heterogeneity of depressive symptoms within the adolescent population and is similar to findings from studies conducted among general adolescents (Dai et al., 2025). Notably, this study focused on adolescents who persistently experienced bullying victimization, a high-risk group, and a similar latent structure was still observed in this group, suggesting that the latent classification of adolescent depressive symptoms may have a certain degree of stability.

However, compared with general adolescent populations, adolescents who persistently experience bullying victimization may differ in the distribution proportions across depressive symptom classes and in their developmental trends. The results of this study showed that from T1 to T2, the proportion of the Low Symptom group decreased from 58.4% to 32.1%, whereas the proportions of the Moderate Symptom group and High Symptom group increased from 30.9% and 10.7% to 45.3% and 22.6%, respectively. This change indicates that the distribution of depressive symptoms in this group showed a tendency to concentrate toward higher levels over time. One possible explanation is that persistent bullying victimization may weaken individuals’ psychological adaptive resources (Östberg et al., 2018), making it more difficult for them to maintain a lower depressive state and thereby leading to a tendency to transition toward moderate-to-high levels of depressive symptoms over time (Winding et al., 2020).

### 4.2. Stability and Changes in Depressive Symptoms

The latent transition analysis (LTA) results indicated that different latent classes of depressive symptoms differed in their temporal stability and transition probabilities. Overall, depressive symptoms showed a tendency to transition from lower to higher levels. Specifically, the High Symptom group showed the highest stability, with approximately 75.8% of individuals remaining in this class at the subsequent measurement. The Moderate Symptom group showed relatively lower stability, with only 50.5% of individuals remaining in the original class, while 41.9% further transitioned to the High Symptom group. The Low Symptom group showed the lowest stability, with only 48.8% of individuals remaining at the original level and more than half (51.2%) transitioning to the Moderate Symptom group. This finding differs from previous studies that examined transitions in depressive symptoms among children and adolescents (G. Li et al., 2023; Liang et al., 2023; Shore et al., 2018). This difference may be because the sample in the present study consisted of children who persistently experienced bullying victimization, who may face higher levels of social stress, making depressive symptoms more likely to remain at higher levels (Shore et al., 2018).

### 4.3. Influence of Physical Activity, Sleep Duration, and Substance Use on Adolescent Depression Transitions

In this study, we examined the effects of health behaviors, including physical activity, sleep duration, and substance use, on transitions in adolescent depressive states. The results confirmed that these three covariates were indeed important risk-related factors for depressive transitions among adolescents who persistently experienced school bullying victimization.

First, sufficient physical activity and sleep both significantly reduced the probability of individuals transitioning from a low or moderate depressive state to a higher depressive level, while also increasing the likelihood that some individuals transitioned from a higher depressive state to a lower depressive state. This suggests that physical activity and sleep duration may have protective effects in the development of depressive symptoms among adolescents who persistently experience bullying victimization. One possible explanation is that, for adolescents persistently exposed to school bullying, physical activity provides a relatively positive channel for emotion regulation and social interaction, thereby buffering the negative psychological impact of bullying experiences to some extent and reducing the risk of further worsening depressive symptoms (Kandola et al., 2019). Adequate sleep may also exert protective effects on the development of depressive symptoms through multiple mechanisms. On the one hand, sleep helps maintain normal regulatory functions of the nervous system and promotes the balance of emotion-related neurotransmitters, thereby stabilizing individuals’ emotional states (Goldstein & Walker, 2014). On the other hand, sleep plays an important role in emotional information processing and psychological recovery, helping individuals integrate and regulate negative emotional experiences more effectively (Tempesta et al., 2018). For adolescents who persistently experience school bullying, good sleep may enhance their emotion regulation capacity and stress-coping ability, thereby buffering the negative psychological impact of bullying experiences to some extent and reducing the risk of further worsening depressive symptoms.

Second, substance use behavior significantly increased the probability of individuals transitioning from a low or moderate depressive state to a higher depressive level, while also reducing the likelihood that some individuals transitioned from a higher depressive state to a lower depressive state. This result suggests that substance use may serve as an early behavioral risk marker for subsequent unfavorable transitions in depressive symptom development among adolescents who persistently experience school bullying victimization. Existing evidence indicates that substance use among adolescents may be associated with poorer emotion regulation capacity, higher tendencies toward problem behaviors, and lower levels of psychosocial adjustment (Rodríguez-Ruiz & Espejo-Siles, 2025; Shadur & Lejuez, 2015). In other words, adolescents who report substance use may simultaneously face more individual and environmental risks and may have relatively insufficient resources for coping with stress and regulating negative emotions. In the context of persistent school bullying victimization, these risk factors may further weaken their emotional adaptation capacity, making them more likely to transition from low or moderate depressive symptom states to higher depressive symptom states or to remain in higher depressive symptom states.

Overall, based on latent transition analysis models across two time points (T1 and T2), this study examined the change patterns of latent depressive symptom states among adolescents who persistently experienced school bullying victimization and further analyzed the associations between health behaviors at T1, including physical activity, sleep duration, and substance use, and subsequent depressive state transitions. The findings provide new evidence for understanding the heterogeneity and dynamic developmental processes of depressive symptoms among adolescents who persistently experience bullying victimization. Based on these findings, schools, families, and communities should establish a coordinated support system. While health behavior promotion and mental health education among adolescents should be strengthened, continued efforts should also be made to advance school bullying prevention and intervention, thereby reducing the risk of mental health problems at the source. Meanwhile, special attention should be given to adolescents with insufficient physical activity, inadequate sleep duration, or substance use behavior, and more targeted psychological support, health behavior guidance, and stress-coping resources should be provided. This study revealed the characteristics of depressive symptom state transitions among adolescents who persistently experienced bullying victimization and their related behavioral factors, which may provide a reference for developing adolescent mental health protection strategies and support systems.

## 5. Limitations

Although this study obtained several meaningful findings, some limitations should be noted.

1. The follow-up period of this study was 12 months, and the relatively limited time span may not fully reflect the long-term developmental trajectories of depressive symptoms in adolescents, thereby limiting the generalizability of the findings to some extent. Future studies could extend the follow-up period and increase the number of measurement waves to more precisely capture dynamic changes in depressive symptoms across different developmental stages.
2. This study mainly focused on the effects of health behavior factors, including physical activity, sleep duration, and substance use, on transitions in depressive states, and the range of variables was relatively limited. Future studies could further include multidimensional factors such as family environment, peer relationships, social support, and individual psychological traits to more comprehensively explore the mechanisms underlying transitions in adolescent depressive symptoms.
3. The data in this study were mainly derived from self-reports, which may be affected by factors such as social desirability effects or recall bias, thereby introducing certain measurement bias. Future studies could combine clinical interviews, teacher or parent reports, and objective measurement indicators to validate the findings using multiple methods and improve the reliability of the conclusions.

## 6. Conclusions

This study found that depressive symptoms among adolescents who persistently experienced school bullying victimization could be classified into three latent profiles: Low Depressive Symptoms, Moderate Depressive Symptoms, and High Depressive Symptoms. During the 12-month follow-up period, the overall sample showed a trend of developing from lower to higher levels of depressive symptoms, indicating that the mental health status of adolescents who persistently experience school bullying victimization deserves attention and that targeted interventions are urgently needed. In addition, the covariate analysis showed that health behavior factors, including physical activity, sleep duration, and substance use, were closely associated with the development of depressive symptoms. This suggests that strengthening the monitoring and intervention of health behaviors among adolescents who persistently experience bullying victimization may be an important pathway for improving their mental health. Therefore, in mental health interventions for adolescents who experience school bullying victimization, attention should be paid not only to the bullying experience itself but also to the potential role of health behavior factors in the development of depressive symptoms.

## Figures and Tables

**Table 1:** Descriptive statistics and correlational analysis (N = 785).

	Variable	M (SD)/*n* (%)	1	2	3	4	5	6	7	8	9	10	11	12	13
1	Age, years	13.92 ± 1.69													
2	Gender, male	478 (60.9%)													
3	T1_depressed affect	0.73 ± 0.69			-										
4	T1_positive affect	1.54 ± 0.89			0.222 ***	-									
5	T1_somatic complaints	0.74 ± 0.57			0.753 ***	0.126 **	-								
6	T1_interpersonal problems	0.60 ± 0.74			0.613 ***	0.110 **	0.516 ***	-							
7	T2_depressed affect	0.95 ± 0.63			0.704 ***	0.170 ***	0.504 ***	0.416 ***	-						
8	T2_positive affect	1.81 ± 0.83			0.113 *	0.713 ***	0.088 *	0.083 *	0.344 ***	-					
9	T2_somatic complaints	0.95 ± 0.57			0.403 ***	0.094 *	0.622 ***	0.295 ***	0.708 ***	0.348 ***	-				
10	T2_interpersonal problems	0.84 ± 0.76			0.299 ***	0.103 ***	0.243 ***	0.660 ***	0.509 ***	0.290 ***	0.502 ***	-			
11	Physical activity, adequate	352 (44.8%)			−0.093 *	−0.121 *	−0.098 *	−0.103 *	−0.379 ***	−0.318 ***	−0.395 ***	−0.292 ***	-		
12	substance use, yes	413 (52.6%)			0.316 ***	0.115 *	0.309 ***	0.246 ***	0.425 ***	0.273 ***	0.415 ***	0.299 ***	−0.249 ***	-	
13	Sleep time, adequate	340 (43.3%)			−0.183 ***	−0.131 **	−0.181 ***	−0.107 *	−0.428 ***	−0.303 ***	−0.445 ***	−0.296 ***	0.696 ***	−0.295 ***	-

Note. T1 = Time 1; T2 = Time 2; * *p* < 0.05. ** *p* < 0.01. *** *p* < 0.001.

**Table 2:** Fit statistics for across-time measurement invariance testing.

	Model Tested	χ^2^	df	CFI	TLI	RMSEA	[90% CI]	ΔCFI
Depressive symptoms (4 subdimensions)	Configural invariance	802.2382	327	0.9738	0.9695	0.0431	[0.039, 0.047]	-
	Metric invariance	778.7290	343	0.9760	0.9734	0.0403	[0.037, 0.044]	<0.01
	Scalar invariance	878.5962	365	0.9717	0.9705	0.0424	[0.039, 0.046]	<0.01
	Error variance invariance	891.7411	383	0.9719	0.9722	0.0412	[0.038, 0.045]	<0.01

Note. χ^2^ = chi-square; df = degrees of freedom; CFI = comparative fit index; TLI = Tucker–Lewis index; RMSEA = root mean square error of approximation; 90% CI = 90% confidence interval; ΔCFI = change in CFI.

**Table 3:** Model fit information and model selection criteria for latent profile analyses at two time points.

	Model	AIC	BIC	ABIC	Entropy	*p*-Value of VLMR	*p*-Value of BLRT	Proportion (%)
T1	1	6855.9	6893.23	6856.09	-	-	-	100
	2	5969.17	6029.83	5969.65	0.883	<0.001	<0.001	77.9/22.1
	**3**	**5644.99**	**5728.98**	**5645.89**	**0.846**	**<0.001**	**<0.001**	**58.4/30.9/10.7**
	4	5551.98	5659.29	5553.43	0.861	<0.05	<0.001	52.7/32.3/11.1/3.9
	5	5449.25	5579.89	5451.4	0.853	0.379	<0.001	53.7/7.3/25.8/6.3/7
T2	1	6653.22	6690.54	6653.4	-	-	-	100
	2	5776.96	5837.62	5777.43	0.802	<0.001	<0.001	64.4/35.6
	**3**	**5466.12**	**5550.1**	**5467.01**	**0.809**	**<0.001**	**<0.001**	**32.1/45.3/22.6**
	4	5429.65	5536.96	5431.1	0.778	0.054	<0.001	35.5/31.3/10.8/22.4
	5	5349.16	5479.8	5351.31	0.838	<0.05	<0.001	21.7/16.4/8/36.6/17.2

Note. The final model is in bold. AIC = Akaike information criterion; BIC = Bayesian information criterion; ABIC = sample-size-adjusted BIC; VLMR *p* = Vuong–Lo–Mendell–Rubin test; BLRT *p* = bootstrap likelihood ratio test.

**Table 4:** Profile differences on the CES-D dimensions and total score across two time points.

		Depressed Affect		Positive Affect		Somatic Complaints		Interpersonal Problems	
		M (SD)	F	M (SD)	F	M (SD)		M (SD)	F
T1	LS	0.31 (0.01)	1275.48 ***	1.41 (0.04)	22.82 ***	0.41 (0.01)	560.16 ***	0.23 (0.02)	263.34 ***
	MS	1.05 (0.02)		1.68 (0.04)		1.03 (0.02)		0.97 (0.04)	
	HS	2.23 (0.04)		1.96 (0.07)		1.78 (0.05)		1.70 (0.09)	
T2	LS	0.33 (0.02)	1148.50 ***	1.27 (0.05)	107.04 ***	0.42 (0.02)	832.92 ***	0.25 (0.02)	660.90 ***
	MS	0.96 (0.02)		1.99 (0.03)		0.97 (0.02)		0.92 (0.03)	
	HS	1.78 (0.03)		2.20 (0.05)		1.68 (0.03)		1.82 (0.04)	

Note. LS = Low Symptom, MS = Moderate Symptom, HS = High Symptom. *** *p* < 0.001.

**Table 5:** Unconditional latent transition probabilities from T1 to T2.

		T2		
		LS-P	MS-P	HS-P
T1	LS-P	0.488	0.512	0.00
	MS-P	0.076	0.505	0.419
	HS-P	0.118	0.124	0.758

Note. T1 = Time 1; T2 = Time 2; LS-P = Low Symptom Profile; MS-P = Moderate Symptom Profile; HS-P = High Symptom Profile.

**Table 6:** Covariate effects on latent transition probabilities from T1 to T2.

Influencing Factor	Latent State	LS-P			MS-P			HS-P		
		B (SE)	OR	95% CI	B (SE)	OR	95% CI	B (SE)	OR	95% CI
T1–T2 Physical activity	LS-P	Ref			−1.796 *** (0.366)	**0.166**	**[0.081, 0.340]**	−4.842 *** (0.675)	**0.008**	**[0.002, 0.030]**
	MS-P	1.796 *** (0.366)	**6.026**	**[2.941, 12.346]**	Ref			−3.046 *** (0.489)	**0.048**	**[0.018, 0.124]**
	HS-P	NA			3.046 *** (0.489)	**21.031**	**[8.065, 54.841]**	Ref		
T1–T2 substance use	LS-P	Ref			1.715 *** (0.226)	**5.557**	**[3.568, 8.654]**	1.764 *** (0.359)	**5.836**	**[2.887, 11.794]**
	MS-P	−1.715 *** (0.226)	**0.180**	**[0.116, 0.280]**	Ref			0.048 (0.341)	1.049	[0.538, 2.047]
	HS-P	NA			−0.048 (0.341)	0.953	[0.489, 1.860]	Ref		
T1–T2 Sleep time	LS-P	Ref			−2.035 *** (0.261)	**0.131**	**[0.078, 0.218]**	−4.599 *** (0.422)	**0.010**	**[0.004, 0.023]**
	MS-P	2.035 *** (0.261)	**7.652**	**[4.588, 12.763]**	Ref			−2.564 *** (0.378)	**0.077**	**[0.037, 0.161]**
	HS-P	NA			2.564 *** (0.378)	**12.988**	**[6.191, 27.245]**	Ref		

Note. Bold values indicate statistically significant results; LS-P = Low Symptom Profile; MS-P = Moderate Symptom Profile; HS-P = High Symptom Profile. NA indicates transitions that were not estimated because the corresponding pathways were theoretically implausible and therefore constrained to zero in the latent transition model. *** *p* < 0.001.

## Data Availability

The data presented in this study are available from the corresponding author upon reasonable request. The data are not publicly available due to privacy and ethical restrictions related to the protection of participants, as the dataset was derived from the Hubei Provincial Student Health Surveillance Program involving minors.
